# Understanding the relationship between stress, distress and healthy lifestyle behaviour: a qualitative study of patients and general practitioners

**DOI:** 10.1186/1471-2296-14-166

**Published:** 2013-11-01

**Authors:** Suzanne H McKenzie, Mark F Harris

**Affiliations:** 1 School of Medicine and Dentistry, James Cook University, Townsville, Australia; 2 Centre for Primary Health Care and Equity, School of Public Health and Community Medicine, University of New South Wales, Sydney, Australia

## Abstract

**Background:**

The process of initiating and maintaining healthy lifestyle behaviours is complex, includes a number of distinct phases and is not static. Theoretical models of behaviour change consider psychological constructs such as intention and self efficacy but do not clearly consider the role of stress or psychological distress. General practice based interventions addressing lifestyle behaviours have been demonstrated to be feasible and effective however it is not clear whether general practitioners (GPs) take psychological health into consideration when discussing lifestyle behaviours. This qualitative study explores GPs’ and patients’ perspectives about the relationship between external stressors, psychological distress and maintaining healthy lifestyle behaviours.

**Methods:**

Semi-structured telephone interviews were conducted with 16 patients and 5 GPs. Transcripts from the interviews were thematically analysed and a conceptual model developed to explain the relationship between external stressors, psychological distress and healthly lifestyle behaviours.

**Results:**

Participants were motivated to maintain a healthy lifestyle however they described a range of external factors that impacted on behaviour in both positive and negative ways, either directly or via their impact on psychological distress. The impact of external factors was moderated by coping strategies, beliefs, habits and social support. In some cases the process of changing or maintaining healthy behaviour also caused distress. The concept of a threshold level of distress was evident in the data with patients and GPs describing a certain level of distress required before it negatively influenced behaviour.

**Conclusion:**

Maintaining healthy lifestyle behaviours is complex and constantly under challenge from external stressors. Practitioners can assist patients with maintaining healthy behaviour by providing targeted support to moderate the impact of external stressors.

## Background

The process of initiating and maintaining healthy lifestyle behaviours is complex, includes a number of distinct phases and is not static [1]. Theoretical models of behaviour change consider psychological constructs such as intention and self efficacy but do not clearly consider the role of psychological or emotional distress [2-4]. Although the transtheoretical model describes feelings such as being unhappy or disappointed as ‘critical situations’ tempting people to perform unwanted behaviour, these and other processes of change have been relatively neglected by researchers [1].

Psychological distress results from the internal response to external stressors. When individuals encounter a stressful situation they perform an internal and usually subconscious appraisal of the situation and depending on their beliefs, sense of control, situational demands or constraints, resources such as social network/s, perceptions of harm and coping styles, they will either develop positive or negative feelings and associated physiological changes, with longer term sequelae being somatic health/illness, morale/well being and social functioning [5]. Psychological distress is also referred to as stress or emotional distress [6]. These terms are used interchangeably in the literature to refer to negative emotional states.

Stress has been identified as a barrier to the uptake of behaviour change [7] although some studies suggest it may need to reach a threshold level before impacting on behaviour [8,9]. High levels of psychological distress are associated with psychopathology such as depression and anxiety [10]. Depressed individuals may be less likely to start a lifestyle program and more likely to drop out, however there is limited evidence that anxiety impacts on either [11]. A range of external factors have also been identified as important influences on behaviour change although it is not clear how these interact with or relate to internal factors such as distress. A recent framework for understanding behaviour suggests that internal and external factors both impact on motivation which in turn influences behaviour and that they also impact directly on behaviour [12]. However the actual processes of these interactions have not been explored.

General practitioners (GPs) in Australia are encouraged to discuss lifestyle behaviours with patients [13]. Structured general practice based interventions addressing lifestyle behaviours and other vascular risk factors have been demonstrated to be feasible and effective [14-16]. However there are few studies that also examine their impact on patient’s psychological health. It is not clear whether GPs take psychological health or well-being into consideration when discussing lifestyle behaviours, nor whether they perceive any un-intended consequences from their interventions. There is some evidence that patients who participate in group programs addressing lifestyle risk factors may have higher psychological distress than those who don’t [17] however little is known about how patients and their GPs address distress in the context of changing or maintaining lifestyle behaviours.

Therefore this study aimed to explore GPs’ and patients’ perspectives about the relationship between external factors or stressors, psychological distress and maintaining healthy lifestyle behaviours. The following research questions were addressed:

1. How are external stressors and distress perceived to influence healthy lifestyle behaviour?

2. Do the participants feel that making healthy lifestyle behaviour changes result in changes to psychological distress?

3. What factors do participants report as moderating the relationships between external stressors, distress and behaviour change?

Improved understanding of these perspectives and processes will inform the practice of promoting healthy lifestyle behaviours and may influence the design and conduct of future general practice based interventions addressing the behavioural risk factors for vascular disease.

## Methods

### Context

This study was conducted in conjunction with the Health Improvement and Prevention Study (HIPS), a cluster randomised controlled trial (RCT) of a complex intervention to reduce cardiovascular risk factors in general practice patients aged 40–69 years [14]. Patients included in the trial had at least one risk factor for cardiovascular disease. The intervention included a health check and referral to a lifestyle modification program consisting of individual allied health visits and a group program. The trial was conducted in two rural and three urban divisions of general practice in NSW, Australia (local primary care support organizations).

### Sample

A purposive sampling strategy aimed to recruit a representative range of patients and general practitioners from the intervention arm of the HIPS study. Recruitment and data collection occurred in one rural and one urban division of general practice, 3 to 6 months following the HIPS intervention.

### Recruitment of patients

Patients had completed the Kessler Psychological Distress Scale (K10) as part of their baseline questionnaire for the HIPS study. Using this scale, patients were stratified as Low (K10 score, 10–15), Medium (K10 score, 16–29) and High (K10 score, 30–50) Distress [10] and then gender. In each division of general practice, fifteen males and fifteen females from each distress group were randomly selected using a random number generator and sent an invitation letter, a participant information sheet outlining the purpose of the interview and a consent form. If less than 15 were in a group then all were included. In total 67 females and 61 males were invited to participate. The first two males and females from each distress group in each division who returned their consent form and contact phone number were included in the study.

### Recruitment of general practitioners

Sixteen GPs from the intervention group were sent an invitation letter, information sheet and a consent form. The interviewer (SM) then telephoned each GP to discuss the information and organise an interview time if they consented to the study. All GPs who consented were included.

### Ethics

The study was approved by the University of New South Wales Human Research Ethics Committee. Participants gave written informed consent.

### Data collection

Semi-structured telephone interviews began with introductions and the aim of the project. Interviews with patients aimed to explore their perspectives about the roles of stress and distress in maintaining healthy behaviours and acting on advice from their doctor about how to improve their health. Interviews with the GPs aimed to explore their perspectives about the role of stress and distress in managing cardiovascular risk factors, particularly lifestyle change. Patients and GPs were encouraged to describe examples from their own experience. Table 1 provides the questions from the interview schedules.

**Table 1:** Questions from the semi-structured interview schedules

**Patients**	**General practitioners**
Think about the relationship between stress and your ability to make a lifestyle change. How easy is it to make a change when you are stressed?	Do you think stress impacts on some patients’ ability to make lifestyle changes? How?
Prompt- Some people who are stressed, are not able to think about making any changes to their lifestyle- what do you think about this? Can you give me some examples from your experience?	Prompt- some patients are unable to change their lifestyle when they are stressed- can you give me any examples of this?
Prompt- Others become stressed when they try to make a change. Have you had this experience? What do you think about this?	Prompt- What do think is the effect of lifestyle change on patients’ level of stress- do they become more or less distressed if they make a change; why do you think that happens?
Prompt- some people feel better in themselves if they make a lifestyle change (e.g. stop smoking). Have you had this experience? Tell me about it.	
Tell me about your recent health check with your doctor -what happened during the consultation; did the doctor suggest any changes to your lifestyle or medications:	Thinking about one of the patients who visited you for a health check, can you describe the consultation to me? What were the main factors that influenced your decisions about managing the patient?
Prompt- Discuss each issue brought up by the patient- e.g. If advised to increase physical activity then ask- what role does your general well being play in your ability to become more active; does being stressed make a difference to your activity levels?	Prompt- Would you give me some examples of how the consultation or your management might by influenced by a patient’s stress or distress?
Did the doctor make you aware of any risk factors for poor health that you were not already aware of? How did this make you feel- prompts: stressed/ concerned/very anxious/down or depressed; not any different. Has your response changed over time since the health check- e.g. Become more or less stressed/anxious?	Do you think that some patients become stressed as a result of being told they are at risk of cardiovascular disease- if yes can you give me an example from your practice?
Did your doctor refer you to any services to help you tackle your risk factors? If yes, then explore the patient’s perspectives about the effectiveness of the services if they attended and the role of stress in this process; e.g. Did attending the group program help relieve any anxiety you had about your risk factors. Did it help you with other aspects of your life?	How have you found conducting the health checks for the HIPs study? Have you referred any high risk patients to the Change program (individual and group visits)? What influenced you to refer some patients and not others? What role did psychological factors such as patient distress or stress play in your decision whether to refer to the Change program or other services?
If the doctor suggested referral to other services but patient did not take these up- explore reasons why.	
What are your thoughts about stress and its impact on diabetes, heart attacks and strokes? Do you think there is a relationship- if yes in what way; if not why not; Is long term stress more or less important than short term stress?	Based on your experience - what do you think about the relationship between mental health and diabetes or cardiovascular disease?

Interviews were conducted by SM who is a general practitioner. They were audio recorded and then transcribed verbatim. Notes taken at the time of interview were included in the analysis.

### Data analysis

Data analysis was conducted in QSR NVIVO 8 (QSR international, Melbourne, Australia) using thematic content analysis [18,19]. Transcripts and notes were read and re-read to identify initial categories and segments of the interviews were coded according to the categories. Categories were modified as further data were collected and analyzed. Identification of the categories came from the data and was influenced by the literature and background reading. The themes that emerged from the data were consistent with the categories from the theoretical data. Data collection and analysis continued until data saturation was achieved. Throughout the analysis, SM and MH discussed and agreed on the interpretation of data.

The study adheres to the RATS guidelines on qualitative research (http://www.biomedcentral.com/ifora/rats).

## Results

### Participant characteristics

Interviews were carried out with 16 patients and 5 GPs. Interviews ranged from 15–25 minutes.

The demographic characteristics of the patients and practitioners were representative of the participants in the HIPs study. The mean age of patients was 56 with a range 46–64 years. There were 8 females and 8 males from both the low and medium distress groups. Patients from the high distress groups did not consent to the study. The majority (n = 11) were born in Australia and spoke English at home (n = 12). 12 were employed, three retired and 1 unemployed. 14 had completed a higher qualification.

The GPs (2 female) were all in full time practice, three in rural practice and had an average of 20 years experience in general practice. All worked in group practices.

Analysis of the data resulted in three main themes: motivation for healthy behaviour, impact of external stressors and the role of psychological distress. A conceptual model was developed from the data to illustrate the relationship between external factors or stressors, psychological distress and healthy lifestyle behaviour.

#### Motivation for healthy behaviour

Patients and GPs were motivated to make or encourage healthy behavioural choices to improve physiological measures such as hypertension or hypercholesterolaemia. They were also motivated by a desire to reduce the risk of cardiovascular disease or diabetes. Cardiovascular disease in family members strongly influenced patients to address their own risk factors. Some were also motivated to change their lifestyle to reduce distress as this was linked directly to health outcomes.

“*he drinks too much and he sort of doesn’t eat the right food. He eats on the run and, and he’s stressed and I think oh you’re a heart attack waiting to happen*” [Rural Female 1, Low Distress]

This patient went on to describe her motivation to maintain her healthy diet to avoid ending up like her work colleague described in this quote. Participants were generally determined to maintain a healthy lifestyle; however they all described episodes where they faced challenges.

#### Impact of external stressors

Patients used the term “stress” to describe their internal feelings of distress and attributed this stress to a range of factors or external stressors that were generally beyond their control. They also described external factors that directly impacted on behaviour but did not cause distress.

One of the commonly mentioned external stressors was the pressure of work on themselves or family and friends. They felt that poor lifestyle behaviours were the result of this stress.

*“an incident at work which.. caused him a great deal of stress …and he was quite fit and trim and very proud of his health and then I noticed in him a, general decline …and regression in his health to the point now where he smokes and he’s put a lot of weight on”* [Urban Male 1, Low Distress]

Other external stressors were economic pressure, family responsibilities, social pressure and inter-current health problems. The impact of these on behaviour change varied however they were generally considered as direct barriers to healthy behaviour or as causing so much distress that this in turn resulted in unhealthy choices.

A woman under considerable pressure from work and family responsibilities described the emotional impact.

*“I do get stressed at times when I’m faced with a lot of deadlines …and a lot of things to do and family at home to look after and elderly parent and, and that sort of thing …”* [Urban Female 2, Low Distress]

She went on to say that this stress then reduced her ability to maintain a regular walking program despite knowing that this would be good for her health.

Another patient was describing how he had incorporated a healthy lifestyle approach into his routine. He struggled to do regular physical activity and he ate poorly when he was unwell with lymphoma, however now he felt well and able to make healthy lifestyle choices.

“*I have very few stressors in my life now. I mean the only one was getting Non-Hodgkin’s Lymphoma …and the chemotherapy but that was for many years that was the only stress type of thing”* [Rural Male 1, Low Distress]

One participant talked about when she went out and was offered cake that she did not want to eat. She found this stressful however her pre-planned strategy helped her to cope with the situation.

*“People try to force things on you to justify the fact that they want to have it …so we had to talk about this and how very stressful that is …first of all say “no thank you” and they say “oh go on one won’t hurt” …and I just said look it’s not good for my weight it, it makes me sick”* [Female, Urban 1, Medium Distress]

Another patient described wanting to increase her exercise intensity as her GP had suggested this would be good for her health but she believed this would have an adverse effect on her blood pressure and this belief caused her some distress:

*“possibly I should try and increase the exercise types that I do …into a little bit more energetic I suppose, to get my heart rate going but increase my blood pressure and that, I find that scary”* [Urban Female 2, Medium Distress]

The negative impact of external stressors on healthy behaviour was not always attributed to stress. In some cases it was merely a matter of choice or commitment.

A patient described a personal commitment to healthy eating and despite pressures of social situations and his wife who was not as strict with her dietary choices.

*“It’s not like it I don’t feel like it’s (food choices) ever out of my control”* [Urban Male 2, Low Distress]

Another said:

*“the only thing that upsets the routine as far as the exercise goes just what happens at.. at work but I travel away each day to work and sometimes stay away so …things like that that puts you out of a routine*” [Rural Male 1, Medium distress]

Social situations where food and drinks were offered often resulted in unhealthy choices but this was not attributed to stress.

*“I think in social things we can make the wrong choices …Of what we’re eating and or drinking.. And I don’t think it’s always stress”* [Rural Female 2, Medium Distress]

#### Role of psychological distress

All participants felt that distress could impact on behavioural choices in a negative way. The concept of a threshold level of distress was evident in the data with patients and GPs describing a certain level of stress required before it influenced behaviour.

*“if you are stressed or depressed or anxious and someone asks you to stop smoking I think that is just asking too much of somebody, they can only cope with one thing at a time*” [Female GP2]

“*If their depressed they’re not going to initiate much, if their tired all the time from their anxiety symptoms they’re less likely to change much”* [Male GP3]

Therefore GPs tended to address psychological health as a separate issue to cardiovascular disease prevention and felt that mental health issues had to be managed prior to suggesting any lifestyle modifications such as a change in diet or smoking cessation.

*“By the time you are addressing their cardiovascular risk factors you have already dealt with their mental health issues*” [Female GP1]

Patients were less clear about the level of distress required to negatively impact on behaviour. For example, a patient recalled *“If I’m uptight or a little bit thingy or whatever and if there happens to be any chips in the cupboard …I’ll eat them”* [Rural Female 2, Medium Distress]. Another one said, “*you’re probably more inclined to let yourself go I think if you’re stressed*” [Urban Male 2, Low Distress], when referring to healthy lifestyle behaviours.

GPs could see that issues they identified as barriers to change could cause distress and this needed to be discussed in the context of behaviour change. Female GP2 felt that it was important to talk about making time to exercise. If this was not planned then trying to incorporate exercise into an already busy schedule would have a negative impact on the patient’s mood.

*“time particularly is a big barrier that you may pick up and discuss further” and if this is not addressed then it can cause the patient some degree of “stress”*. [Female GP2]

The influence of behaviour on distress was generally described in positive terms. If exercise was increased or weight loss achieved through reducing food intake then this resulted in feeling better or happier.

*“the few kilos that I wanted to lose I’ve lost very, very easily and … I do feel better for it”* [Urban Female 2, Low distress]

Participants described a feedback loop where a change in health status such as an improvement in blood pressure or reduction in cholesterol levels, attributed to healthy lifestyle changes, resulted in an elevated mood and therefore less distress.

*“I changed my lifestyle and in doing so my blood pressure is contained to the point where as long as I’m not stressed my blood pressure doesn’t go up*” [Urban Female 2, Medium Distress]

A female GP described patients who had successfully reduced their cardiovascular risk factors through lifestyle changes; and as a result their self esteem had improved. With that their mood also improved.

*“They are usually so proud and pleased with themselves; it is very good for their self esteem*” [Female GP1]

GPs described strategies such as suggesting small incremental changes and setting personal goals when dealing with anxiety related to changing behaviour.

*“Some people say I can’t do it doctor, so I say you do it bit by bit; they are anxious because they don’t realize they can do that because they have been in a habit for 50 years and suddenly they have to change things”* [Male GP1]

Patients also recognized that setting and achieving goals was an important strategy in the process of behaviour change: *“If you see results, you aspire to continue and, maybe get to a goal or weight or whatever”* [Urban Male 2, Low distress]

However, even if no actual change in outcomes was described, the process of maintaining a healthy lifestyle was part of a general approach to overall well being.

*“I respect my body… and I know there’s good and there’s bad *(in reference to food choices)* and…I try and take a moderate line… I have very few stressors in my life now”* [Rural Male 1, Low distress]

One man (Rural Male 2, Low Distress) described how he moved from the capital city to a rural area and was enjoying regular physical activity on his farm, eating a healthy vegetarian diet and doing regular meditation. He continued to have a high cholesterol level but felt he was doing all he could for his overall health.

#### Conceptual model

**Figure 1:**
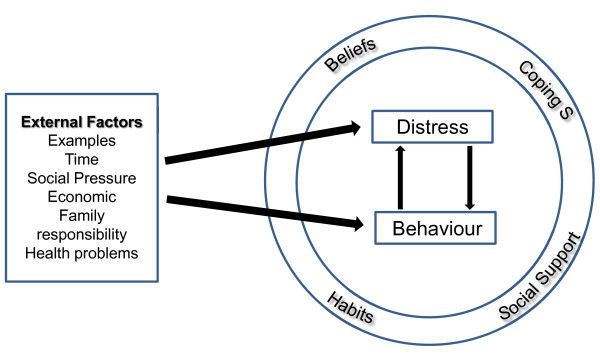
The relationship between external factors, psychological distress and healthy lifestyle behaviour: a conceptual model.

The impact of external stressors on distress or directly on behaviour varied amongst participants and also varied depending on individual circumstances at the time of the stressor. As illustrated in Figure 1, external stressors could impact directly on behaviour choices or via their impact on psychological distress. The impact seemed to be moderated by individual coping strategies, beliefs, habits, and social support. One or several moderators might facilitate or mitigate the impact of an external stressor.

Coping strategies included pre-planned responses, distraction, and avoidance of difficult situations. The common belief amongst participants was the importance of reducing cardiovascular risk through healthy lifestyle choices and this was a strong motivator for maintaining healthy lifestyle behaviours. Many participants described exercise routines that had become habits along with strategies such as checking food labels which had also become habits. Social support was highlighted by many patients as a key moderator. This could work in positive ways through encouragement and direct support for healthy behaviour or against via promotion of unhealthy food choices or creating barriers to physical activity. The positive or negative impact of the various moderators changed frequently and depended on the combination presenting itself at any particular time.

## Discussion

The participants’ account of their attempts to maintain ideal behavioural choices in the context of external stressors and internal distress is consistent with the transactional model of stress and coping [5] and the transtheoretical model of behaviour change [4].

The participants in this study were in the action and maintenance phases of the transtheoretical model [4]. However they described their lifestyle behaviour as a fluid process, one that changed frequently depending on the circumstances and what their feelings and beliefs were at that moment. The interactions between internal distress and behavioural choices were complex and were moderated by beliefs, coping styles and strategies; and social support. Their descriptions were consistent with the transactional model of stress and coping which outlines the moderating effects of coping strategies, personal beliefs and social support on the relationship between external stressors and internal/psychological distress.

Participants perceived that once a threshold level of distress is reached it interacts in a complex way with other processes of change. The transtheoretical model includes processes of change but these have been relatively neglected by researchers [1]. These are strategies that people use when trying to change their behaviour or in protecting their current behaviour from relapse and include the identification and expression of emotions regarding the problem behaviour, appraising values in respect to the behaviour, access and use of social support; and individual commitment to changing the behaviour [4]. The participants in our study described the role of these processes in moderating the relationship between external stressors and psychological distress.

This study has provided a deeper understanding of the complex processes and interactions in maintaining healthy lifestyle behaviours. Distress is only one of many processes that impact on, or can be impacted by behaviour. While the participants in this study regarded it as part of the process of maintaining healthy behaviour, their emphasis was on controlling environmental triggers, social support and appraising their beliefs or values concerning the behaviour.

The psychological distress of the patients participating in the intervention group of the HIPs study was significantly reduced compared to those in the control group although it was not moderated by a change in behaviour [20]. However behavioural processes are difficult to capture using quantitative methods [21]. Our study has explored these processes and found that the HIPs health check and lifestyle modification program reinforced the beliefs and strategies that patients and practitioners were already implementing. This and the role of social support described by the participants in this study add to our understanding of the mechanisms through which the HIPs intervention reduced psychological distress.

Group programs addressing lifestyle behaviours have been demonstrated to be effective but the mechanisms for this effect have often not been described [14,22,23]. Therefore, future interventions in general practice addressing lifestyle behaviours should consider including group processes and tailored individual support addressing the factors described by the participants in this study. New frameworks for characterizing and designing behaviour change interventions could incorporate the individual factors described in this study and link these to intervention functions and policies [12].

The demographic characteristics of patients and GPs; and distress levels of the patients, were representative of the trial participants and the interpretation of the data was conducted with rigor by two researchers. However our findings may not be representative of all patients and practitioners. The majority of patients who participated in this study had completed a higher qualification so our findings may not be applicable to a wider population group. There were few patients who had high psychological distress in the HIPS study and none consented to participate in the interviews, again limiting our findings to those with low to medium psychological distress. The general practitioners who participated were interested in discussing psychological health in the context of behaviour change and their views may not represent all GPs. The telephone interviews were a pragmatic choice based on limited resources but may have resulted in a loss of richness to the data as non-verbal cues may have been missed. The GP interviewer potentially influenced the participants’ accounts as they may have been more likely to give answers that were acceptable to a colleague in the case of the GPs and to a doctor in the case of the patients. The triangulation of our findings, with those from the HIPS study has strengthened our findings.

## Conclusion

Maintaining healthy lifestyle behaviours is complex and constantly under challenge from external stressors. External stressors impact both on behaviour and distress however the impact is moderated by controlling environmental triggers, social support and appraising beliefs or values concerning the behaviour. Our findings are consistent with theoretical models of behaviour change and we have considered the role of mild to moderate psychological distress in this context. Practitioners can assist patients with maintaining healthy behaviours by providing targeted support to moderate the impact of “stress”. Important moderators include developing specific coping strategies for individual stressors; building strong social support for healthy lifestyle choices and identifying unhelpful habits and beliefs.

## Competing interests

Authors declare that they have no competing interest in the conduct of this study.

## Authors’ contributions

Both authors contributed to the conception and design of the study and interpretation of data. SM conducted the interviews. SM wrote the first draft of the manuscript. Both authors contributed to revising the manuscript and approved the final draft.

## Pre-publication history

The pre-publication history for this paper can be accessed here:

http://www.biomedcentral.com/1471-2296/14/166/prepub
